# Ten‐year survival and pattern of recurrence in patients with locally recurrent rectal or sigmoid cancer undergoing resection

**DOI:** 10.1111/codi.17226

**Published:** 2024-12-05

**Authors:** J. N. Wiig, Vegar Johansen Dagenborg, Stein Gunnar Larsen

**Affiliations:** ^1^ Section for Abdominal Cancer Surgery, Norwegian Radium Hospital, Department for Surgical Oncology Oslo University Hospital Oslo Norway; ^2^ Institute for Cancer Genetics and Informatics Oslo University Hospital Oslo Norway

**Keywords:** 10 year follow‐up, locally recurrent rectal cancer, multimodal treatment, surgery

## Abstract

**Aim:**

The aim of this work is to report actual overall survival (AOS) at 5 and 10 years after multimodal treatment for locally recurrent rectal or sigmoid cancer (LRRC) and the importance of local re‐recurrence (reLRRC) and distant metastases for AOS.

**Method:**

All patients resected for LRRC at a single centre between years 1990 and 2007 were included. Resections were based on images taken after neoadjuvant treatment. Patients were prospectively followed up for 5 years. After a minimum of 10 years, the records of referring hospitals were analysed.

**Results:**

A total of 224 patients underwent resection. At 5 and 10 years 33% and 17%, respectively, had survived. Median survival was 38 months [interquartile range (IQR) 62 months]. Patients with complete resections had 5‐ and 10‐year survival of 56% and 28%, respectively, versus 22% and 11% for those with microscopic remaining tumour; none with macroscopic remains survived beyond 4 years. Median survival was 71 months (IQR 106 months), 33 months (IQR 35 months) and 15 months (IQR 17 months), respectively. With a median survival of 123 months (IQR 80 months), the 54 patients without recurrence had 5‐ and 10‐year survival of 69% and 59%, respectively. The independent predictor of survival was R‐stage. Of the 197 patients who had radical resection, 83 developed reLRRC and 108 distant metastases. ReLRRC appeared at a median of 18 months (IQR 21 months) and distant metastases at 12 months (IQR 21 months). Lung metastases were the most common form of distant disease.

**Conclusion:**

More than 5 years postoperatively the mortality from cancer was substantial. Most metastases appeared not to be secondary to reLRRC. Planning surgery from pretreatment images might reduce reLRRC.

**What does this paper add to the literature?**

For surgically managed locally recurrent rectal cancer, there are several studies reporting 3‐ and 5‐year outcomes. Data on longer‐term outcomes are limited, with only one previous study reporting data at 10 years. We found that even after 5 years there was a high rate of recurrent cancer with a consequent impact on survival. Metastases, most frequently in the lungs, potentially contributed to death in half of patients with local re‐recurrence in less than 20%. A complete resection was associated with improved survival.

## INTRODUCTION

The introduction of total mesorectal excision for the resection of a primary rectal cancer has been shown to reduce the frequency of local recurrence from 20%–30% to 5%–10% [1]. The inclusion of neoadjuvant radiotherapy (RT)/chemoradiotherapy (CRT) for locally advanced rectal cancer further reduces the frequency of local recurrence [2, 3]. Locally recurrent rectal cancer (LRRC) is associated with poor quality of life, with pain, faecal incontinence, haematochezia, fistula, urinary obstruction or incontinence and uraemia [4]. Despite the reduction in the overall incidence of recurrence, LRRC remains an important clinical problem.

LRRC was considered inoperable until the mid‐1970s, when results from the Mayo Clinic gave rise to optimism [5]. Today it is accepted that salvage surgery for LRRC, in combination with RT/CRT, can offer local control and prolong survival for some patients [6, 7, 8, 9, 10, 11, 12, 13, 14, 15]. Studies have reported frequencies of locally re‐recurrent cancer (reLRRC) and metastases at 3–5 years following salvage LRRC surgery [6, 7, 8, 9, 10, 11, 12, 13, 14, 15, 16, 17, 18, 19, 20, 21, 22, 23]. Given that only one study followed LRRC patients beyond 5 years [24], the longer‐term natural history of surgically treated LRRC remains understudied.

This study aims to explore the longer‐term survival and pattern of re‐recurrence after resection for LRRC at a single centre.

## METHOD

### Patients

The Norwegian Radium Hospital is a tertiary referral centre for the treatment of LRRC, with nearly all patients referred from other hospitals. Patients with LRRC who had surgery between September 1990 and July 2007 were included. These patients had initial primary cancers located within the rectum or sigmoid (defined as within 16 cm or 16–30 cm from the anal verge, respectively). Only patients without pre‐ or intraoperatively diagnosed distant metastases were included. Age and comorbidity were relative contraindications to surgical resection. All LRRCs were biopsy‐confirmed adenocarcinoma and located below the pelvic brim or in the perineum. Patients were evaluated by a multidisciplinary team of surgeons, oncologists and radiologists. Preoperative local staging was performed by rigid sigmoidoscopy and, initially, CT. From 2001, MRI mostly replaced CT. Abdominal CT or ultrasound, and X‐ray or CT of the chest were performed, with endorectal ultrasound in retroprostatic cancers. Restaging was undertaken 4 weeks after completion of CRT. All patients had complete colonoscopy perioperatively. Recurrent tumours were divided between anastomotic (recurrence adjacent to the anastomosis) predominantly centred within the bowel wall or perirectal (mostly located outside the bowel).

### RT/CRT

Thirty patients were not irradiated due to previous RT. Otherwise RT was performed as previously described [8]. Briefly, 46 Gy in 2‐Gy fractions was applied to the pelvis preoperatively. From 1996, 2 × 2 Gy was added to the tumour region. Between 1990 and 1996 intraoperative RT to the tumour bed was applied (12–20 Gy). This was found to be ineffective and was terminated [25, 26].

### Chemotherapy

Neoadjuvant CRT (NaCRT) with an intravenous or peroral 5‐fluorouracil (FU)‐leucovorin regimen was given after 2003 to patients with bulky tumours adherent to the pelvic sidewall or central pelvic organs [2]. Some patients had other chemotherapy regimens: three patients had FU‐leucovorin and oxaliplatin (FLOX) [3] and three had NaCRT and chemotherapy given with local, pelvic hyperthermia [27]. Patients with reLRRC or metastases had 5‐FU regimens according to national guidelines. Adjuvant chemotherapy was not given routinely after salvage surgery.

### Surgery

Resection radicality was planned on restaging images 4 weeks after RT/CRT. Surgery was undertaken 10 weeks after termination of RT/CRT. *En bloc* resection of the tumour and involved adjacent organs was attempted. A frozen section was performed in some cases where resection margins were doubtful and re‐resection was then performed immediately in fewer than five cases of a tumour‐involved margin. In cases with a definite response after neoadjuvant treatment the resection was less extensive than considered necessary from the preneoadjuvant images. An open pelvic cavity was filled with a voluminous greater omentum. In later years, major perineal defects were reconstructed with a myocutaneous flap (vertical rectus abdominis flap, gluteus maximus flap or extended gluteus maximus flap). The operations involved a surgeon with a gastrointestinal interest, urologists, plastic‐ or orthopaedic surgeons as appropriate. The surgical procedures were classified as ‘central’ (including pelvic floor and anterior pelvic organs), ‘pelvic wall’ (including pelvic wall or sacrum) or ‘combined’ resections.

### Follow‐up

Patients had outpatient clinic appointments every 3 months for 2 years, and thereafter biannually until 5 years. Each visit included rigid sigmoidoscopy where appropriate, CT scan of the pelvis, CT or ultrasound of the abdomen, CT or X‐ray of the lungs and blood samples. Most surviving patients had a colonoscopy approximately 5 years postoperatively.

### Data collection

The preoperative variables collected were gender, location of primary tumour, primary operation, Dukes stage, time from primary operation to first documented evidence of LRRC, a diagnosis due to symptoms at routine follow‐up and location of LRRC. The treatment variables collected were type of salvage operation (central, pelvic wall resection or combined), completeness of resection (R‐stage; defined as R0 = free margin of >1 mm, R1 = free margin of ≤1 mm and the few cases of re‐resection and R2 = remaining macroscopic tumour) and preoperative RT.

Follow‐up more than 5 years after a salvage operation was based on symptoms and included the use of CT, ultrasound or X‐rays. Studied variables within 5 years postoperatively were prospectively entered in the department's database. After 1 July 2017 the medical records of the referring hospitals were analysed for reLRRC or distant metastases. Dates of death were retrieved from the National Cancer Registry of Norway.

### Statistics

Variables were described using median with interquartile range (IQR) and percentages. Actual overall survival (AOS) was recorded for all patients and defined from salvage surgery to date of death. Differences between categorical variables, as percentage AOS at 5 and 10 years postoperatively, were tested by chi‐square with Pearson's modification.

The Kaplan–Meier method with log‐rank test compared differences in survival. Multivariate analysis (Cox backward Wald) was performed on prognostic variables achieving *p* < 0.05 in univariate testing. All tests were two‐sided, pooled or pairwise. Missing values were not substituted in the tests. The statistics were performed with SPSS v.25. *p* < 0.05 was considered statistically significant. Analysis of expected mortality in a normal Norwegian population was performed at The National Cancer Registry of Norway [28].

## RESULTS

**FIGURE 1:**
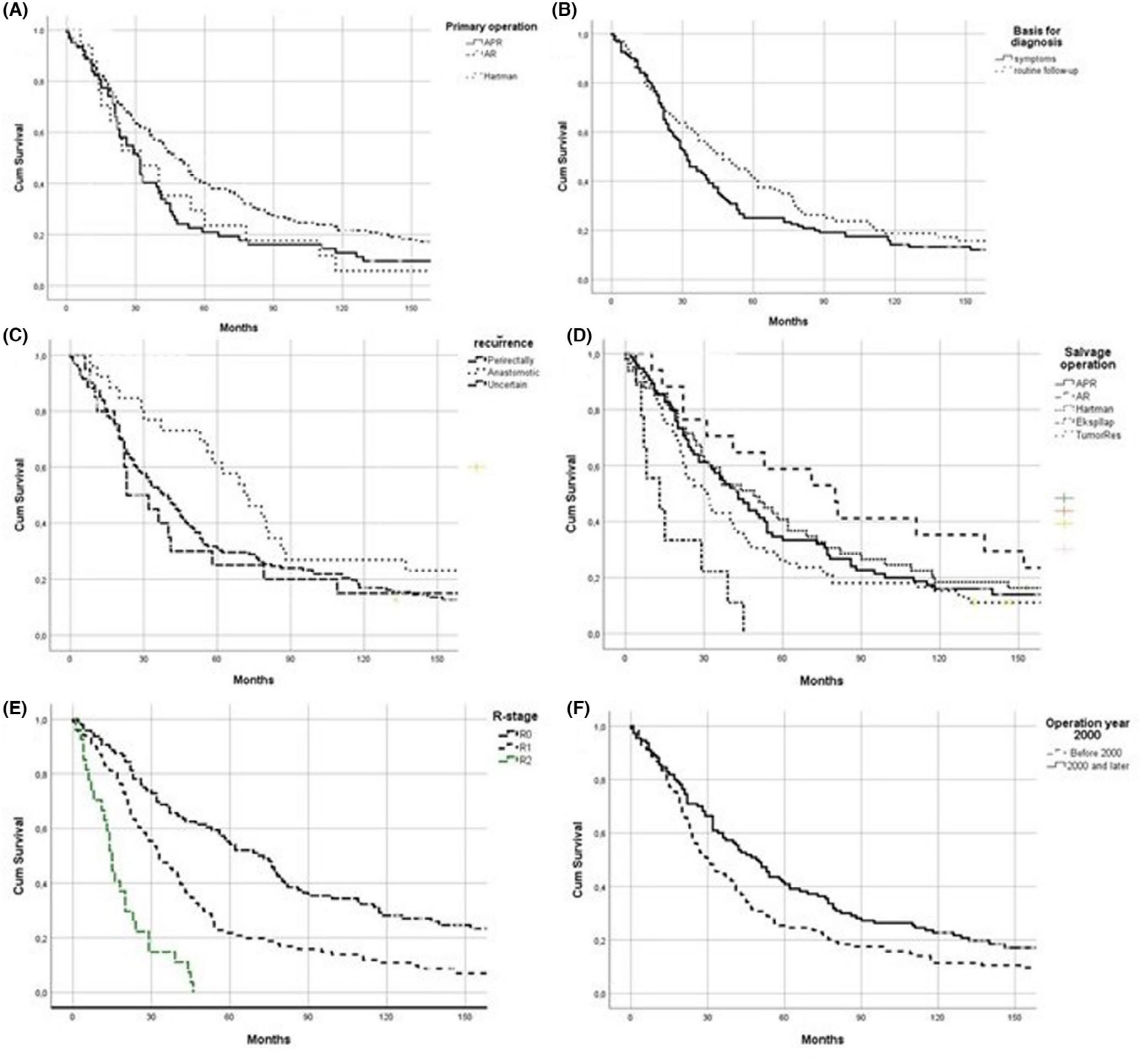
Kaplan–Meier survival curves of univariate statistically significant prognostic factors of all resections after operation for locally recurrent rectal/sigmoid cancer: (A) primary operation (224 patients); (B) basis for diagnosis (201 patients); (C) origin of recurrence (224 patients); (D) salvage operation (224 patients); (E) R‐stage of the salvage operation (224 patients); (F). AR, anterior resection; Expllap, exploratory laparotomy; Hartman, Hartmann's operation; PR, abdominoperineal resection; TumorRes, tumour resection.

The study included 224 patients with a median age at salvage operation of 68 years (IQR 12 years) (Table 1). Twelve patients were older than 80 years, and 64% patients were male. The primary tumour location was in the rectum in 196 patients (88%) with 28 (12%) in the sigmoid. The primary operation was anterior resection (AR) in 134 (60%) patients, abdominoperineal resection (APR) in 62 (28%), Hartmans's resection in 17 (8%), sigmoid resection in 6 (3%) and missing in 5. Prognostic variables associated with surgery for the primary tumour are shown in Table 1. The AOS for the 224 patients at 5 and 10 years was 33% (75 patients) and 17% (38 patients), respectively, with a median AOS of 38 months (IQR 62 months). The median AOS for 134 AR patients was 47 months (IQR 82 months) versus 31 months (IQR 31 months) for 62 APR patients (*p* = 0.016). The AOS of 40% and 21%, respect at 5 years was significantly better for AR (*p* = 0.008) but not at 10 years (22% vs. 13%) (p=0.148) (Figure 1A). The presence of symptoms was associated with outcomes, with the 80 (40%) asymptomatic patients having a better 5‐year AOS (41%) versus 25% for the 120 (60%) symptomatic patients (*p* = 0.042) (Figure 1B). The difference was not significant at 10 years (41% vs. 25%) (*p* = 0.624). Median AOS was 47 months (IQR 75 months) and 32 months (IQR 50 months), respectively (*p* = 0.058). Similarly, the AOS of the 26 (13%) patients with nastomotic recurrence (62%) was significantly better at 5 years than the AOS for the 134 (69%) perirectal recurrences (32%) (*p* = 0.004) (Figure 1C), but this was not significant at 10 years (27% vs. 17%) (*p* = 0.126). Median AOS for anastomotic recurrences was 71 months (IQR 102 months) versus 38 months (IQR 64 months) (*p* = 0.027).

**TABLE 1:** Basic prognostic variables after surgery for the primary rectal cancer (*N* = 224).

Total	*n* (%)	*n* at 5 years (AOS%)	*χ* ^2^ *p*‐value	*n* at 10 years (AOS%)	*χ* ^2^ *p*‐value	Multiariate *p*‐value	Median AOS (months) (IQR)	Log‐rank *p*‐value
Age (years) (mean)	68							
Total	224	75 (33)		38 (17)			38 (62)	
Gender			0.342		0.100			0.092
Male	144 (64)	45 (31)		20 (14)			36 (60)	
Female	80 (36)	30 (38)		18 (23)			41 (95)	
Location			0.872		0,346			0.216
Rectum	196 (88)	67 (34)		35 (18)			40 (61)	
Sigmoid	28 (12)	8 (32)		3 (11)			24 (72)	
Dukes stage			0.795		0.729			0.364
A		9 (39)		4 (17)			36 (75)	
B	65 (39)	22 (34)		11 (17)			40 (73)	
C	79 (47)	23 (32)		10 (13)			31 (62)	
Missing	57							
Operation			0,172		0.368	0.543		0.010
AR	134 (60)	54 (40)	*0.008	29 (22)	* 0.148		47 (134)	*0.016
APR	62 (28)	13 (21)		8 (13)			31 (31)	
Sigmoid res.	6 (3)	2 (33)		0			22 (67)	
Hartmann	17 (8)	5 (29)		1 (6)			32 (55)	
Other	5 (2)						7	
Diagnosis			0.042		0.624	0.805		0.058
No Symptoms	80 (40)	33 (41)		15 (19)			47 (75)	
Symptoms	120 (60)	30 (25)		17 (14)			32 (50)	
Other	1							
Missing	23							
Origin of recurrence			0.012		0.155	0.239		0.119
Anastomotic	26 (13)	16 (62)	**0.004	7 (27)	**0.126		71 (102)	**0.027
Perirectal	142 (69)	45 (32)		24 (17)			38 (64)	
Perineal	3 (2)	2 (67)		1 (26)			126	
Uncertain	20 (10)	5 (25)		3 (15)			23 (55)	
Other	16 (8)	3 (19)		2 (13)			26 (37)	
Missing	17							
LRRC delay			0.393		0.219			0.098
All	224						21.5 (25)	
<12 months	63 (28)	24 (38)		11 (18)			42 (64)	
12–23 months	59 (26)	17 (29)		11 (19)			29 (62)	
24–35 months	69 (31)	20 (29)		9 (13)			37 (40)	
≥36 months	33 (15)	14 (42)		7 (21)			50 (87)	

*Note*: *n* (%), actual percentage survivors (AOS%) at 5/10 years; median actual overall survival (AOS) in months with interquartile range (IQR); *χ* ^2^, chi‐square test;

Abbreviations: AR, anterior resection; APR, abdominoperineal resection; Hartmann, Hartmann's operation; LRRC, locally recurrent cancer; LRRC delay, time from primary rectal cancer surgery to recurrent disease; res., resection.

* *p* for AR versus APR.

** *p* for anastomotic recurrence versus perirectal recurrence.

### Treatment variables

Treatment variables are shown in Table 2. The 17 patients (8%) having salvage AR operations had better median AOS [80 months (IQR 134 months)] than those with resection of separate pelvic tumours [*n* = 72 (32%), 32 months (IQR 49 months)] (*p* = 0.036) (Figure 1D).

**TABLE 2:** Perioperative prognostic variables after surgery for recurrent rectal cancer (*N* = 224).

Total	*n* (%)	*n* at 5 years (AOS%)	*χ* ^2^ *p*‐value	*n* at 10 years (AOS%)	*χ* ^2^ *p*	Cox multivariate *p*‐value	Median AOS (IQR)	Log‐rank *p*‐value
Salvage operation			0.039		0.363	0.548		0.003
AR	17 (8)	10 (59)	*0.276	6 (35)	*0.904		80 (134)	*0.133
APR	75 (33)	35 (26)		12 (16)			43 (66)	
Hartmann	49 (22)	20 (41)		9 (18)			49 (84)	
Tumour res.	72 (32)	19 (26)		11 (15)			31 (49)	**0.036
Expl. lap.	9 (4)	0		0			13 (28)	
Other	2 (1)	0		0			42	
Pelvic res.			0.535		0.627			0.392
Central only	39 (17)	15 (39)		7 (18)			41 (88)	
Pelvic wall only	59 (26)	22 (37)		13 (22)			37 (75)	
Combined	77 (34)	21 (27)		11 (14)			32 (57)	
Rectal only	49 (22)	17 (35)		7 (14)			40 (63)	
R‐stage			<0.001		<0.001	<0.001		<0.001
R0	96 (43)	53 (56)		27 (28)			71 (106)	
R1	101 (45)	22 (22)		11 (11)			33 (35)	
R2	27 (12)	0		0			15 (17)	
RT performed			0.219		0.568			0.781
Yes	194 (87)	62 (32)		34 (18)			37 (61)	
No	30 (13)	13 (43)		4 (13)			42 (71)	

*Note*: *n* (%); actual percentage survivors (AOS%) at 5/10 years; median actual overall survival (AOS) in months with interquartile range (IQR); *χ* ^2^, chi‐square test; Cox, Cox multivariate analysis.

Abbreviations: AR, anterior resection; APR, abdominoperineal resection; Expl. lap, exploratory laparotomy; Hartmann, Hartmann's operation; res., resection; RT, radiotherapy.

* *p* for AR versus APR.

** *p* for AR versus tumour resection.

Completeness of resection was a strong prognostic factor for survival. AOS for the 96 (43%) patients with R0 resections at 5 and 10 years was 56% (53 patients) and 28% (27 patients), respectively This was better than for the 101 (45%) patients with R1 resection with a 5‐ and 10‐year survival of 22% (22 patients) and 11% (11 patients) (*p* < 0.001). Median AOS for R0 resections was 71 months (IQR 106 months) and 33 months (IQR 35 months) for R1 resections, with no patients who had R2 resections surviving beyond 4 years (*p* < 0.001) for all comparisons (Figure 1E).

Of the 30 (13%) previously irradiated patients (24 had primary rectal and 6 had other pelvic cancers), RT, gender, location of primary tumour, Dukes stage and time to LRRC were not associated with survival in univariate analysis.

In multivariate analysis, completeness of resection (R‐stage) was the only variable associated with prognosis (*p* < 0.001). Multivisceral resections were performed in 179 patients and more details are given in Table 3. Fifty‐nine (26%) patients had resection of the pelvic wall without central resection and 39 (17%) had central resection without pelvic wall resection; 77 (34%) had both resections combined (*p* = 0.535).

**TABLE 3:** Multivisceral resections during surgery for recurrent cancer (*N* = 179).

	*n* (%)
TPE	26 (12)
Bladder	2 (1)
Prostate	2 (1)
Vesicle	71 (32)
Hysterectomy	29 (13)
Vagina	42 (42)
Pelvic wall	136 (61)
Central	116 (52)
Sacrum	9 (4)
Below S2	3 (1)
Below S3	3 (1)
Below S4	3 (1)
Small bowel	45 (20)
Myocutaneous flaps	14 (6)
VRAM	12 (5)
Ext.Glut.Max.	1 (0.5)
Glut.Max.	1 (0.5)

Abbreviations: Ext.Glut.Max., extended gluteus maximus muscle; Glut.Max., gluteus maximus muscle; TPE, total pelvic exenteration; VRAM, vertical rectus abdominis muscle.

### Long‐term outcomes for reLRRC and metastases

Long‐term outcomes for reLRRC and metastases are shown in Table 4. ReLRRC occurred in 83 patients (42%) and distant metastases in 108 (55%), including 45 patients (23%) who developed both reLRRC and distant metastases. Fifty‐four patients (27%) had no evidence of recurrence (noRecurrence) at the end of the study period.

**TABLE 4:** Numbers with local re‐recurrences or metastases in R0/R1 resected patients (*N* = 197).

	*n* (%)	Median months (IQR)	5‐year AOS, *n* (%)	10‐year AOS, *n* (%)	Median AOS (months) (IQR)
reLRRC time to diagnosis	83 (42	1 (12			
Met. time to diagnosis	108 (55)	12 (21)			
reLRRC‐only duration to death	35 (19)	11 (13)			
Met.‐only duration to death	60 (32)	21 (44)			
reLRRC‐only AOS	35	5 (14)	5 (14)	1 (3)	24 (41)
Met.‐only AOS	60	20 (33)	20 (33)	7 (12)	41 (41)
noRecurrence AOS	54 (27)	37 (69)	37 (69)	32 (59)	123 (80)

*Note*: all times are in months.

Abbreviations: AOS, actual overall survival from diagnosis in months; Met., metastases; noRecurrence, no sign of local re‐recurrence or distant metastasis; reLRRC, locally re‐recurrent cancer.

In 13 cases (7%) the reLRRC became apparent before the metastases, and in 11 cases (6%) it was detected simultaneously. By 5 years, 82% of the reLRRCs and 85% of the distant metastases had developed, and at 10 years the figure was 92% for both groups.

The median time from surgery to diagnosis for all 83 reLRRCs was 18 months (IQR 21 months) and 12 months (IQR 21 months) for the 108 metastases.

The median time to death after diagnosis for the 35 reLRRC‐only patients was 11 months (IQR 13 months) and for the 60 metastases‐only patients it was 21 months (IQR 44 months).

**FIGURE 2:**
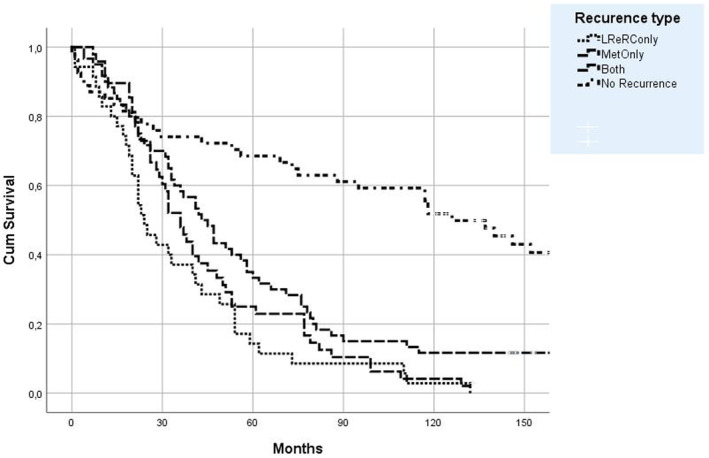
Kaplan–Meier survival curves of local re‐recurrence (LReR), distant metastasis and no recurrence in 197 R0/R1 resected patients. Both, both LReR and distant metastasis in the same patient; LReRConly, only locally re‐recurrent cancer; MetOnly, only distant metastasis; No recurrence, without a diagnosis of recurrent cancer.

The 5‐ and 10‐year AOS for the 35 reLRRC‐only patients was 14% and 3%, respectively, for the 60 patients with metastases‐only it was 33% and 12%, and for the 54 noRecurrence patients it was 69% and 59% (Figure 2). Differences between the noRecurrence and recurrence groups were statistically significant at both 5 and 10 years (*p* < 0.001). For reLRRC‐only versus metastases‐only differences in AOS were statistically significant at 5 years (*p* = 0.029) but not at 10 years.

**FIGURE 3:**
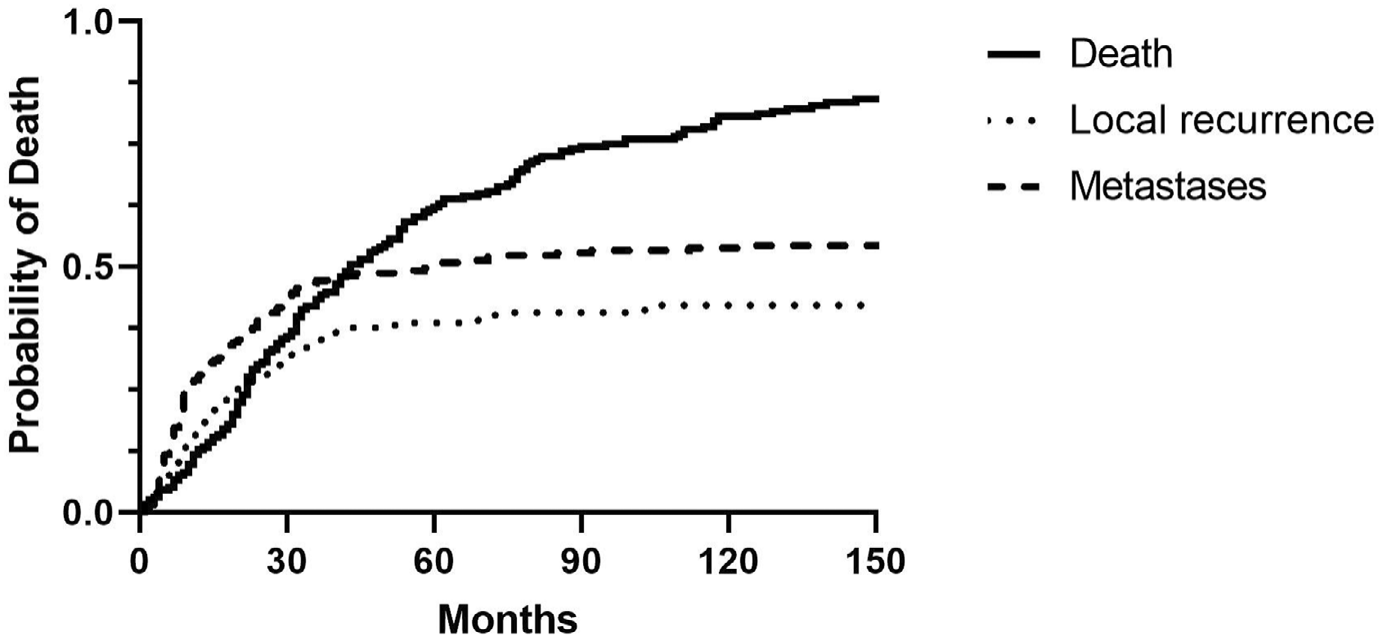
Probability of local re‐recurrence, distant metastasis and death in 197 R0/R1 resected patients.

Median AOS for the 35 patients with reLRRC‐only was 24 months (IQR 37 months) and 41 months (IQR 41 months) for the 60 patients with metastases‐only. This is in strong contrast to the median AOS for the 54 noRecurrence patients who had an AOS of 123 months (142 months) (Figure 3).

Among the 75 patients alive at 5 years, 49% died during the next 5 years. This included 82% of the 17 patients with reLRRC, 69% of the 33 with metastases and 22% of the 37 noRecurrence patients. The observed survival of the noRecurrence patients was 92% (95% CI 71.84–106.32) compared with survival in a ‘normal’ Norwegian population supplied by the Norwegian Cancer Registry (data not shown) [28].

ReLRRC appeared less frequently after R0 than after R1 resection (32% vs. 52%, *p* = 0.006) in contrast to being equally frequent in distant metastases, with 52% for R0 resections and 58% for R1 (*p* = 0.299) (data not shown).

Distant metastases occurred in 108 of 224 patients: lungs (25%), liver (14%), bone (6%), lymph nodes (5%), peritoneum (3%) and brain (2%). A further 9% of patients developed metastases in other organs. Multiorgan development was most common in lungs and liver (10%).

## DISCUSSION

Few data exist concerning the long‐term survival and recurrence rates for patients undergoing surgery for LRRC. This paper presents the 10‐year survival and rates with local re‐recurrence and distant metastases for a cohort of patients who underwent resection. Although the R1 rates were higher than for current cohorts, the 5‐ and 10‐year overall survival rates of all 224 patients were 33% and 17%, respectively, with a median AOS of 38 months. In those patients with an R0 resection, AOS was 56% and 28%, respectively. Completeness of resection was the only independent prognostic variable in multivariate analysis. This has been shown by most [10, 12, 16, 17, 18], but not all [8], authors.

Due to the lack of a generally accepted staging system for LRRC [5, 29] and the difference in classification of R1 resection [30, 31], studies on LRRC are difficult to compare. The new classification gives both fewer R1 resections, superior results for R0 and inferior results for R1. Differences in results could also be due to varying surgical technique, selection of patients with varying tumour size and location [13, 19, 32] and suboptimal pathological evaluation [33].

Long‐term AOS has been reported in a recent study on 46 patients, of whom 80% had R0 resection and no known distant metastasis at the time of surgery [24]. Their 5‐ and 10‐year AOS was 46% and 37%, respectively. Our 10‐year survival is less than reported in that study, although our 5‐year survival is in line with theirs and others (26%–46%) [8, 12, 14, 15, 16, 22, 23].

### Prognostic variables for survival

R0 resection was the only multivariate predictor for long‐term survival. In this historical cohort, the R0 rate was 43%. This is at the lower end for studies that reported rates (between 43% and 67%) [9, 10, 11, 13, 14, 15, 16, 18, 22]. Several factors can influence the ability to achieve a complete resection. Some studies report on R0/R1 operations without stating whether R2 resections were excluded or avoided. Our 56% 5‐year AOS for R0 resections compares well with the results of other studies (31%–62%) [9, 10, 12, 14, 15, 23]. The 5‐year AOS of R1 resections (22%) is similar to other works [9, 14, 15] and statistically significantly better than for R2 resections, suggesting that R1 resections can improve survival. In a recent multicentre study [34] starting from 2004 and including 1210 patients from 26 hospitals, low‐volume hospitals achieved around 50% R0 resections while high‐volume centres improved their R0 rate from around 50% to above 60%. Some hospitals even reported R0 resection rates of around 80% [13, 24, 35]. The multicentre study [34] seems to show a correlation between increasing rates of ‘bone resections’ and improved R0 stage. However, the lack of definition of ‘bone resection’ is problematic. Still, our 4% sacral resection rate was low but corresponds to surgical practice around the year 2000. During this period, the extent of surgical resection was based on images taken 4 weeks after completion of RT. This can underestimate the extent of the tumour and consequently the resection required [36]. To improve R0 resection rates we now generally plan surgery from the images taken before neoadjuvant treatment. Indeed, CRT regimens have changed significantly, which may have led to improvements in longer‐term survival.

The variables primary operation, symptoms at diagnosis, origin of LRRC and salvage operation had a statistically significant influence on survival at 5 years but not at 10 years postoperatively, possibly due to too few patients at risk at 10 years. Anastomotic cancers and cancers having a salvage AR were all small tumours, with a high rate of R0 resections and accordingly better prognosis [7, 32].

Patients without symptoms at diagnosis of LRRC had marginally better AOS than the symptomatic group. MRI imaging at follow‐up might possibly increase the frequency of asymptomatic recurrences [36]. The length of the interval until LRRC did not influence survival. In contrast to others, we did not find a statistical difference comparing central or pelvic side wall resections [17]. The apparent lack of influence on outcome for RT might be due to differences in selection criteria [37].

### Pattern of local and distant recurrences

Our rates of reLRRC (42%) and distant metastases (55%) are similar to previous results for reLRRC (21%–56%) and for metastases (33%–62%) [11, 12, 13, 15, 23]. The reLRRC rate of 31% after R0 surgery could be due to the challenge faced by pathologists in evaluating the R‐stage in bulky tumours that affect multiple organs [5, 33]. The vast majority of reLRRCs and distant metastases were diagnosed within 5 years. This is similar to previous reports [23]. Our survival rate in the noRecurrence group from 5 to 10 years postoperatively was 92% compared with the ‘normal’ Norwegian population. This suggests that there were few cancer deaths in this group, and furthermore that few cancer recurrences were missed in our retrospective follow‐up.

The 6‐month shorter median time to distant metastases compared with reLRRC and the fact that 83% of the metastases appeared without or before reLRRC, as well as the lack of correlation between development of metastases and R‐stage, suggests that the majority of metastases developed from the primary cancer or primary recurrence. In line with an earlier study, and in contrast to primary cancer [38, 39], we found highest rates of metastases in the lungs. This can reflect a difference in venous drainage after the primary

### Limitations

The database only includes those patients who were selected for surgery, and therefore it is difficult to quantify the impact of our selection criteria on longer‐term outcome. The database does not contain data on the involved pelvic compartments. CT was the main diagnostic tool, neoadjuvant chemotherapy was only scarcely applied and re‐irradiation or hyperfractionated irradiation not at all. Follow‐up after 5 years was performed retrospectively. Therefore, the findings of this study may not reflect more current practice.

## CONCLUSION

Even more than 5 years postoperatively the mortality from cancer was substantial. Most metastases appeared not to be secondary to reLRRC. Planning surgery from images taken before chemotherapy treatment might reduce the rate of reLRRC.

## AUTHOR CONTRIBUTIONS

**J. N. Wiig:** Software; conceptualization; methodology; data curation; validation; investigation; formal analysis; supervision; visualization; writing – original draft; writing – review and editing; project administration. **Vegar Johansen Dagenborg:** Software; methodology; validation; formal analysis; resources; writing – review and editing; writing – original draft. **Stein Gunnar Larsen:** Methodology; data curation; investigation; validation; formal analysis; project administration; resources; writing – review and editing; writing – original draft.

## FUNDING INFORMATION

There is no separate funding.

## CONFLICT OF INTEREST STATEMENT

The authors declare no conflicts of interest.

## ETHICS STATEMENT

The regional ethics committee has declared acceptance unnecessary. The patients have consented to inclusion in the database. The data protection officer of the hospital has approved the study.

## Data Availability

The data that support the findings of this study are available on request from the corresponding author. The data are not publicly available due to privacy or ethical restrictions.
